# Supplementary material for: Inhibition of Tyrosyl-DNA Phosphodiesterase 1 by Lipophilic Pyrimidine Nucleosides
Source: Molecules. 2020 Aug 13;25(16):3694. doi: 10.3390/molecules25163694 (PMC7465190; doi:10.3390/molecules25163694)
Supplement: Supplementary file 1 [file molecules-25-03694-s001.pdf]

Inhibition of tyrosyl-DNA phosphodiesterase 1 by lipophilic pyrimidine nucleosides

Alexandra L. Zakharenko[1‡], Mikhail S. Drenichev[2‡], Nadezhda S. Dyrkheeva[1‡], Georgy A. Ivanov[2], Vladimir E. Oslovsky[2], Ekaterina S. Ilina[1], Irina A. Chernyshova[1], Olga I. Lavrik[1,3]* and Sergey N. Mikhailov[2]*

[1]Institute of Chemical Biology and Fundamental Medicine, Siberian Branch of the

Russian Academy of Sciences, 8 Lavrentiev Ave., Novosibirsk, 630090, RussianFederation

[2]Engelhardt Institute of Molecular Biology, Russian Academy of Sciences, 32 Vavilova

Str., 119991, Moscow, Russian Federation

[3]Department of Natural Sciences, Novosibirsk State University, 2 Pirogova Str,.Novosibirsk,630090, Russian Federation

[‡]The authors contributed equally

***** Correspondence: lavrik@niboch.nsc.ru (Lavrik O.I.), smikh@eimb.ru (Mikhailov S.N.); Tel.: (optional; include country code; if there are multiple corresponding authors, add author initials) +7(383) 363-51-95 (Lavrik O.I.), +8-499-135-9733 (Mikhailov S.N.).

General

The solvents and materials were reagent grade and were used without additional purification. Column chromatography was performed on silica gel (Kieselgel 60 Merck, 0.063-0.200 mm). TLC was performed on Alugram SIL G/UV254 (Macherey-Nagel) with UV visualization. Melting points were determined with Electrothermal Melting Point Apparatus IA6301 and are uncorrected. ^{1}H and ^{13}C (with complete proton decoupling) NMR spectra were recorded on Bruker AMX 400 NMR instrument. ^{1}H-NMR-spectra were recorded at 400 MHz and ^{13}C-NMR-spectra at 100 MHz. Chemical shifts in ppm were measured relative to the residual solvent signals as internal standards ($CDCl_3$, ^{1}H: 7.26 ppm, ^{13}C: 77.1 ppm; DMSO-d_6, ^{1}H: 2.50 ppm, ^{13}C: 39.5 ppm). Spin-spin coupling constants (*J*) are given in Hz.The following compounds were prepared according to the methods reported earlier: compound **2g** [Vorbrüggen, Chem.Ber., 1981 – *here and after Refs from the manuscript*], derivatives of pyrimidine-4-one, pyrimidine-2-one, 2-oxo-4-methoxypyrimidine(**5-7**) [Niedballa&Vorbrüggen, J. Org. Chem. **1977**; Vorbrüggen&Bennua, Chem.Ber., **1981**; Vorbrüggen, Acc. Chem. Res. **1995**], 2′,3′,5′-tri-*O*-benzoylcytidine (**8**) [Prasad et al., Bioorg. Med. Chem. **2005**]. The values of the partition coefficient of the compounds between the octanol-water phases (logP) were calculated using the Instant J. Chem.(ChemAxon®) software.

Synthesis of compounds 2a-f

A number of 5-substituted derivatives of 2′,3′,5′-tri-*O*-benzoyluridine was synthesized starting from the corresponding 5-substituted uridines by their direct *O*-benzoylation with benzoyl cyanide according to the procedure elaborated by Prasad an colleagues [Prasad et al., Bioorg. Med. Chem. **2005**, 13, 4467-4472] (**Scheme 1**).

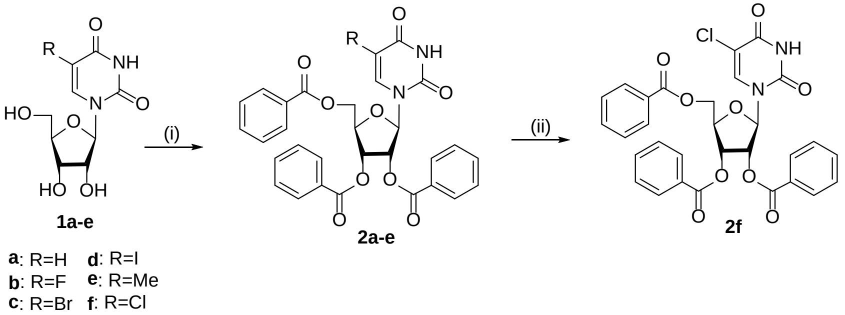

Scheme 1. Synthesis of 5-substituted 2′,3′,5′-tri-*O*-benzoyluridine derivatives.

Reagents and conditions: (i) BzCN/Et_3N, dioxane, r.t., 40 min, 70% (R=H, Me), 68% (R=Br), 56% (R=I); (ii) **2a**, ClNSu/Py, r.t., 15 min, 94%.

The short-time treatment of 2′,3′,5′-tri-*O*-benzoyluridine (**2a**) with *N*-chlorosuccinimide in pyridine at ambient temperature gave pure 2′,3′,5′-tri-*O*-benzoyl-5-chlorouridine (**2f**) with near quantitative yield. 2′,3′,5′-Tri-*O*-benzoyl-5-fluorouridine (**2b**) and 2′,3′,5′-tri-*O*-benzoyl-5-methyluridine (**2e**) were obtained by glycosylation of 5-fluorouracil or thymine correspondingly with an excess of 1-*O*-β-D-2,3,5-tri-*O*-benzoyl-β-D-ribofuranose in the presence of TMSCl and Lewis acids ($SnCl_4$ or TMSOT*f*) according to the earlier elaborated procedures [Mikhailov et al., Curr. Prot. Nucl. Acid Chem. **2006**; Vorbrüggen, Acc. Chem. Res. **1995**]. The presence of benzoyl groups at ribofuranosyl moiety of the synthesized compounds was confirmed by the presence of signals of phenyl protons in the low-field region (8.15-7.30 ppm) and a broad signal of uracil 3NH-group at approximately 12-11.5 ppm in ^{1}H-NMR spectra and characteristic ^{13}C-signals, related to phenyl moieties and three C=O-groups in ^{13}C-NMR spectra. 2′,3′,5′-Tri-*O*-benzoyluridine **2a** was characterized by the presence of signals of CH-protons of uracil residue at position 5 as doublet of doublets with coupling constants $^3J_{5,6}$ = 8.1 Hz, $^4J_{NH,5}$= 2.1 Hz at 5.63 ppm and position 6 as doublet with coupling constant $^3J_{6\text{-}5}$ = 8.1 Hz at 7.87 ppm. For compounds **2c** and **2f** with bromine or chlorine substituents at position 5 of uracil there was a distinct signal of 6CH-proton of uracil residue as a singlet in ^{1}H-NMR spectra. The presence of fluorine atom at position 5 of pyrimidine residue in compound **2b** was confirmed by spin-spin coupling constant between ^{19}F and ^{1}H in ^{1}H-NMR spectra ($J_{6H\text{-}F}$ = 6.5 Hz) and between ^{19}F and ^{13}C in ^{13}C-NMR spectra ($^1J_{6C\text{-}F}$ = 230 Hz). In ^{13}C-NMR of 5-iodouridine derivative **2d**

a strong displacement of a C-5 signal towards strong magnetic field (69.79 ppm) in a comparison with C-5 in uracil analogue (110–100 ppm) was observed, which is characteristic for compounds, containing iodine atoms.

2′,3′,5′-Tri-*O*-benzoyluridine (2a).

To a solution of uridine (1g, 4.7 mmol, 1 eq) in dry dioxane (40 mL), benzoyl cyanide (BzCN) (2.03 g, 15.51 mmol) and triethylamine (2.2 ml, 15.51 mmol) were added in one portionand the reaction mixture was stirred at ambient temperature for 40 min to full dissolving of BzCN. The reaction mixture was then treated with 15 mL of MeOH. The resulting solution was left to stay for 30 min at ambient temperature and then evaporated in vacuum. The residue was co-evaporated with CH_2Cl_2(10 mL). The product was crystallized from CH_2Cl_2 (3 mL). The precipitate was filtered, washed with mixture CH_2Cl_2 (3×2 mL) and dried in vacuum desiccator over P_2O_5 to yield1.84 g (70%) of white crystals. M.p. 153°C. R_f = 0.45 (CH_2Cl_2/EtOH- 99/1, v/v). ^{1}H-NMR (400 MHz, DMSO-d_6): 11.49 (d, ^4J = 2.1 Hz, 1H, NH^3), 8.04 (dd, 2H, ^3J = 8.5 Hz, ^4J = 1.4 Hz, *o*-Bz), 7.93-7.85 (m, 4H, *o*-Bz), 7.83 (d, 1H, $^3J_{6\text{-}5}$ = 8.1 Hz, H-6 Ura), 7.68 – 7.60 (m, 3H, *p*-Bz), 7.52 (dd, 2H, ^3J = 8.5 Hz, ^3J = 6.9 Hz, *m*-Bz), 7.49-7.41 (m, 4H, *m*-Bz), 6.16 (d, $J_{1',2'}$ = 3.6 Hz, 1H, H-1′), 5.97-5.88 (m, 2H, H-2′, H-3′), 5.67 (dd, $^2J_{5\text{-}6}$= 8.1 Hz, ^4J = 2.1 Hz, 1H, H-5 Ura), 4.74 (ddd, $J_{4',3'}$ = 6.2 Hz, $J_{4',5'a}$ = 3.6 Hz, $J_{4',5'b}$ = 5.5 Hz, 1H, H-4′), 4.72 (dd, 1H, $J_{5'a,4'}$ = 3.6 Hz, $J_{5'a,5'b}$ = - 11.8 Hz, H-5′b), 4.64 (1H, $J_{5'b,4'}$ = 5.5 Hz, $J_{5'b,5'a}$ = -11.8 Hz, H-5′b). ^{13}C-NMR (100 MHz, DMSO-d_6): 165.44 (C=O), 164.60 (C=O), 164.57 (C=O), 163.04 (C-4), 150.27 (C-2), 142.20 (C-6), 133.88, 133.78, 133.48, 129.27 (Bz), 129.18 (Bz), 128.69 (Bz), 128.53 (Bz), 128.43 (Bz), 102.24 (C-5), 89.55 (C-1′), 78.74 (C-4′), 73.16 (C-2′), 70.49 (C-3′), 63.62 (C-5′).

2′,3′,5′-Tri-*O*-benzoyl-5-bromouridine (2c).

To a solution of 5-bromouridine (200 mg, 0.62 mmol) in dry dioxane (40 mL), benzoyl cyanide (BzCN) (268 mg, 2.05 mmol) and triethylamine (0.3ml, 2.05mmol) were added in one portion and the reaction mixture was stirred at ambient temperature for 40 min to full dissolving of BzCN. The reaction mixture was then treated with 15 mL of MeOH. The resulting solution was left to stay for 30 min at ambient temperature and then evaporated in vacuum. The residue was co-evaporated with CH_2Cl_2(10 mL) and purified by column chromatography on silica-gel. The product was eluted with

CH_2Cl_2/EtOH = 99/1 (*v/v*) to give 132mg (68%) of **2b** as a white foam.R_f = 0.96 (CH_2Cl_2/EtOH = 99/1, v/v). ^{1}H-NMR (400 MHz, $CDCl_3$): 8.50 (brs, 1H, HN^3), 8.13 (dd, 2H, ^3J = 7.2 Hz, ^4J = 1.4 Hz, *o*-Bz), 7.99 (dd, 2H, ^3J = 7.2 Hz, ^4J = 1.3 Hz, *o*-Bz), 7.94 (dd, 2H, ^3J = 7.2 Hz, ^4J = 1.3 Hz, *o*-Bz), 7.76 (s, 1H, H-6, 5-BrUra), 7.66-7.55 (m, 3H, *p*-Bz), 7.53 (dd, 1H, ^3J = 7.2 Hz, ^3J = 6.4 Hz, *m*-Bz), 7.51 (dd, 1H, ^3J = 7.2 Hz, ^3J = 8.3 Hz, *m*-Bz), 7.42 (dd, 2H, ^3J = 7.2 Hz, ^3J = 8.3 Hz, *m*-Bz), 7.46-7.32 (m, 4H, *m*-Bz), 6.37 (d, 1H, $J_{1',2'}$ = 6.0 Hz, H-1′), 5.89 (dd, 1H, $J_{3',2'}$ = 6.0 Hz, $J_{3',4'}$ = 3.5 Hz, H-3′), 5.73 (t, 1H, $J_{2',1'}$ = $J_{2',3'}$ = 6.0 Hz, H-2′), 4.83 (dd, 1H, $J_{5'a,5'b}$ = - 13.6 Hz, $J_{5'a,4'}$ = 3.9 Hz, H-5′a), 4.76-4.73 (m, 1H, H-4′, overlapping withH-5′b), 4.72 (dd, 1H, $J_{5'b,5'a}$ = - 13.6 Hz, $J_{5'b,4'}$ = 3.4 Hz, H-5′b). ^{13}C-NMR (100 MHz, DMSO-d_6): 165.45 (C=O), 164.55 (C-4), 159.06 (C=O), 149.65 (C-2), 141.38 (C-6), 133.86 (Bz), 133.77 (Bz), 133.49 (Bz), 129.28 (Bz), 129.13 (Bz), 128.72 (Bz), 128.68 (Bz), 129.64 (Bz), 128.52 (Bz), 128.45 (Bz), 96.52 (C-5), 89.26 (C-1′), 78.98 (C-4′), 73.23 (C-2′), 70.25 (C-3′), 63.56 (C-5′).

2′,3′,5′-Tri-*O*-benzoyl-5-iodouridine (2d).

The procedure was analogous to the preparation of **2b**, starting from 5-iodouridine (200 mg, 0.54 mmol).Yield 117 mg (56%) as a white foam. R_f = 0.96 (CH_2Cl_2/EtOH = 99/1, v/v). ^{1}HNMR (400 MHz, DMSO-d_6): 11.86 (s, 1H, HN^3), 8.30 (s, 1H, H-6,5-IUra), 8.02 (dd, 2H, ^3J = 8.4 Hz, ^4J = 1.4 Hz, *o*-Bz), 7.91-7.85 (m, 4H, *o*-Bz), 7.71-7.58 (m, 3H, *p*-Bz), 7.51 (dd, 2H, ^3J = 7.8 Hz, ^3J = 6.9, *m*-Bz), 7.48-7.40 (m, 4H, *m*-Bz), 6.18 (d, 1H, $J_{1',2'}$ = 3.5 Hz, H-1′), 5.97-5.90 (m, 2H, H-2′, H-3′), 4.78-4.71 (m, 1H, H-4′, overlapping withH-5′a), 4.67 (dd, 1H, $J_{5'a,5'b}$ = - 12.0 Hz, $J_{5'a,4'}$ = 3.8 Hz, H-5′a, overlapping withH-5′b), 4.65 (dd, 1H, $J_{5'b,5'a}$ = - 12.0 Hz, $J_{5'b,4'}$ = 5.6 Hz, H-5′b). ^{13}C-NMR (100 MHz, $CDCl_3$): 166.17 (C=O), 165.44 (C=O), 165.40 (C=O), 159.47 (C-4), 149.79 (C-2), 144.05 (C-6), 133.95, 133.90, 133.80, 130.02, 129.93, 129.86, 129.04, 128.68, 128.65 (Bz), 87.74 (C-1′), 81.14 (C-4′), 73.93 (C-2′), 71.49 (C-3′), 69.79 (C-5, 5I-Ura), 63.96 (C-5′).

2′,3′,5′-Tri-*O*-benzoyl-5-methyluridine (2e).

The procedure was analogous to the preparation of **2b**, starting from 5-methyluridine (ribothymidine) (200 mg, 0.77 mmol). Yield 309 mg (70%) as a white foam. R_f = 0.55 (CH_2Cl_2/EtOH = 99/1, v/v). ^{1}H-NMR (400 MHz, DMSO-d_6): 11.46 (s, ^{3}NH), 8.03 (dd, 2H, ^3J = 8.5 Hz, ^4J = 1.4 Hz, *o*-Bz), 7.91

(dd, 2H, ^3J = 8.5 Hz, ^4J = 1.4 Hz, *o*-Bz), 7.87 (dd, 1H, ^3J = 8.5 Hz, ^4J = 1.4 Hz, *o*-Bz), 7.71-7.60 (m, 4H, *p*-Bz+H-6 Thy), 7.52 (dd, 2H, ^3J = 7.6 Hz, ^3J = 8.5 Hz, *m*-Bz), 7.49-7.40 (m, 4H, *m*-Bz), 6.20 (d, $J_{1',2'}$ = 4.2 Hz, 1H, H-1′), 5.96-5.87 (m, 2H, H-2′, H-3′), 4.78-4.73 (m, 1H, H-4′, overlapping withH-5′a), 4.73 (dd, 1H, $J_{5'a,4'}$ = 3.5 Hz, $J_{5'a,5'b}$ = - 12.5 Hz, H-5′a), 4.63 (1H, $J_{5'b,4'}$ = 5.8 Hz, $J_{5'b,5'a}$ = -12.5 Hz, H-5′b), 1.68 (s, 3H, Me). ^{13}C-NMR (100 MHz, DMSO-*d*$_6$): 165.44 (C=O), 164.61 (C=O), 164.55 (C=O), 163.62 (C-4), 150.34 (C-2), 137.03 (C-6), 133.89, 133.79, 133.54, 129.28, 129.18, 129.06, 128.75, 128.68, 128.55, 128.35 (Bz), 110.08 (C-5), 88.38 (C-1′), 78.73 (C-4′), 73.02 (C-2′), 70.56 (C-3′), 63.64 (C-5′), 11.83 (Me).

2′,3′,5′-Tri-*O*-benzoyl-5-chlorouridine (2f)

A solution of 2′,3′,5′-tri-O-benzoyluridine (893 mg, 1.6 mmol) and *N*-chlorosuccinimide (NCS) (686 mg, 5.1 mmol) in dry pyridine (15 mL) waskept at ambient temperature for 15-20 min until greencoloring.The reaction mixture was evaporated in vacuum and co-evaporated with ethyl acetate (10 mL) and methylene chloride(10 mL). The residue was purified by column chromatography on silica-gel. The product was eluted with CH_2Cl_2/EtOH = 99/1 (*v/v*) to give909 mg (94%) of **2f** as white crystals.M.p. = 200°C (dec). R_f = 0.58 (CH_2Cl_2/EtOH - 99/1, v/v). ^{1}H-NMR (400 MHz, DMSO-*d*$_6$): 12.03 (s, 1H,HN^3), 8.25 (s, 1H, H-6, 5Cl-Ura), 8.02 (dd, 2H, ^3J = 7.4 Hz, ^4J = 1.3 Hz, *o*-Bz), 7.88 (dd, ^3J = 7.2 Hz, ^4J = 1.2 Hz, 4H, *o*-Bz), 7.72 – 7.58 (m, 3H, *p*-Bz), 7.57 – 7.36 (m, 6H, *m*-Bz), 6.20 (d, 1H, $J_{1',2'}$ = 2.9 Hz, H-1′), 6.00 – 5.86 (m, 2H, H-2′, H-3′), 4.82 – 4.74 (m, 1H, H-4′, overlapping withH-5′a), 4.69 (dd, 1H, $J_{5'a,5'b}$ = - 12.2 Hz, $J_{5'a,4'}$ = 3.4 Hz, H-5′a, overlapping withH-5′b), 4.67 (dd, 1H, $J_{5'b,5'a}$ = -12.2 Hz, $J_{5'b,4'}$ = 5.9 Hz, H-5′b). ^{13}C-NMR (100 MHz, DMSO-*d*$_6$): 165.48 (C=O), 164.57 (C-4), 158.94 (C=O), 149.47 (C-2), 138.94 (C-6), 133.89 (Bz), 133.80 (Bz), 133.52 (Bz), 129.33 (Bz), 129.30 (Bz), 129.16 (Bz), 128.73 (Bz), 128.71 (Bz), 128.66 (Bz), 128.54 (Bz), 128.47 (Bz), 107.97 (C-5), 89.22 (C-1′), 79.03 (C-4′), 73.27(C-2′), 70.26 (C-3′), 63.58 (C-5′).

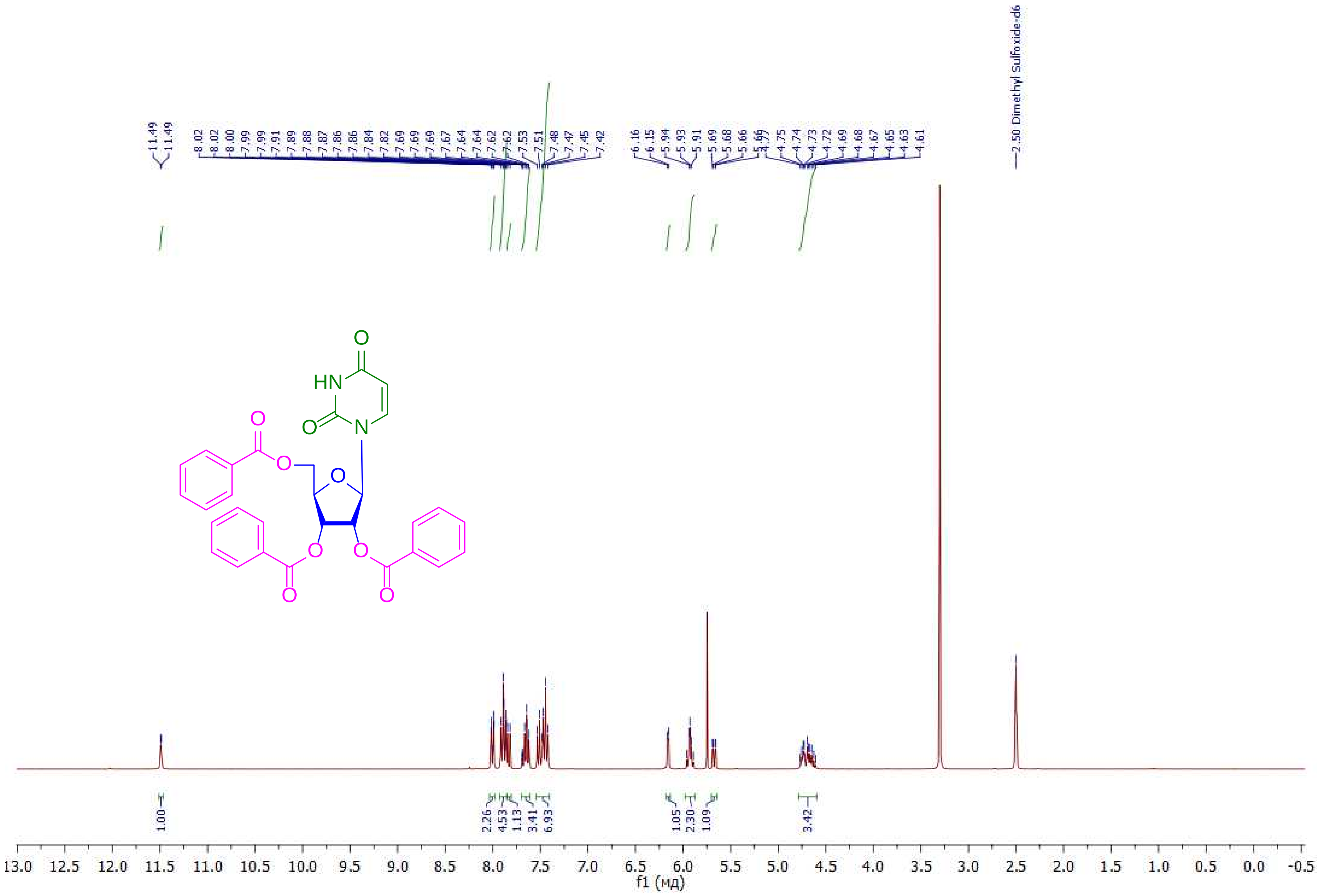

Fig.1. ^{1}H-NMR-spectrum (400 MHz) of 2′,3′,5′-tri-O-benzoyluridine in DMSO-d_6 at 298 K

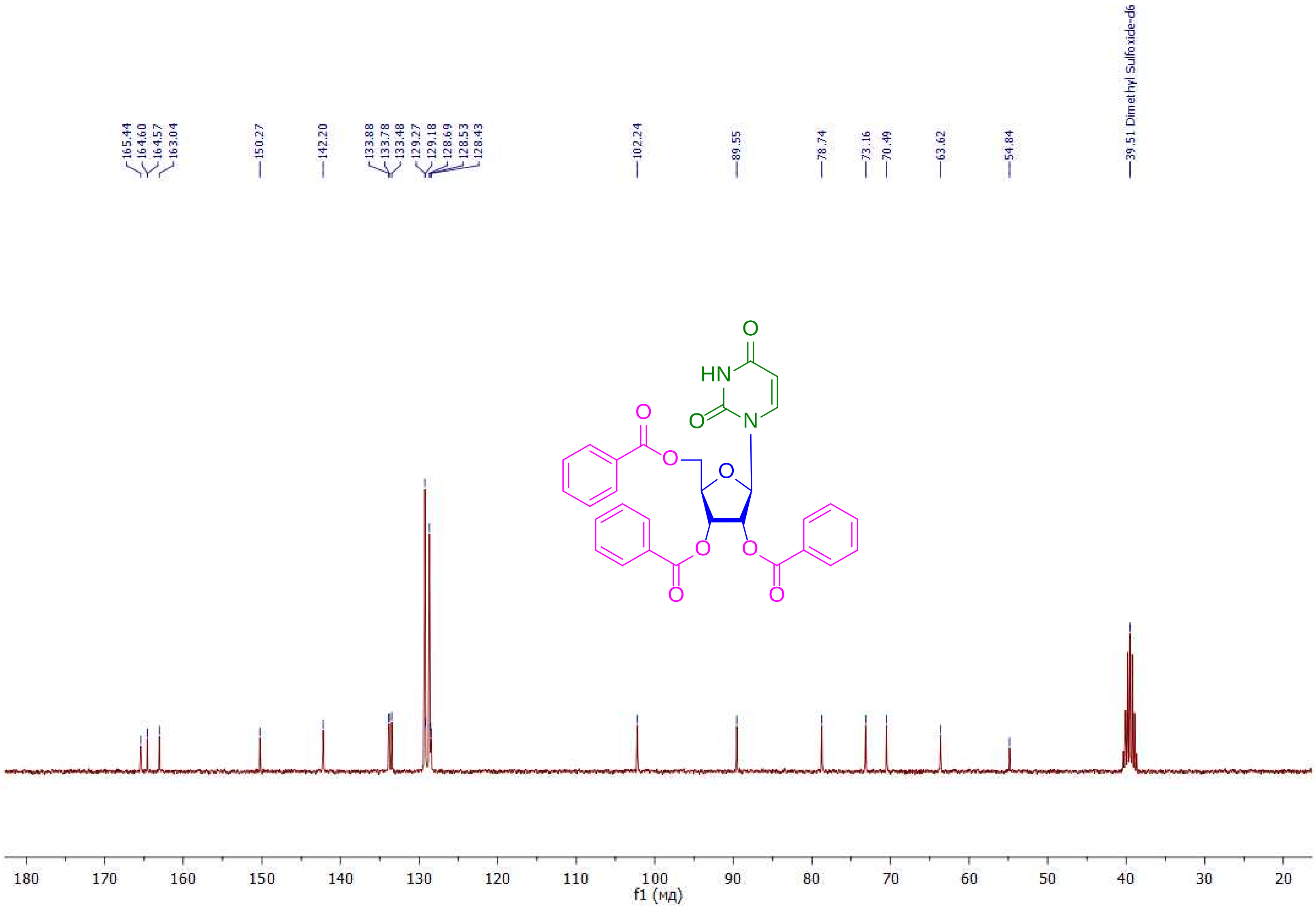

Fig.2. ^{13}C-NMR-spectrum (400 MHz) of 2′,3′,5′-tri-O-benzoyluridine in DMSO-d_6 at 298 K

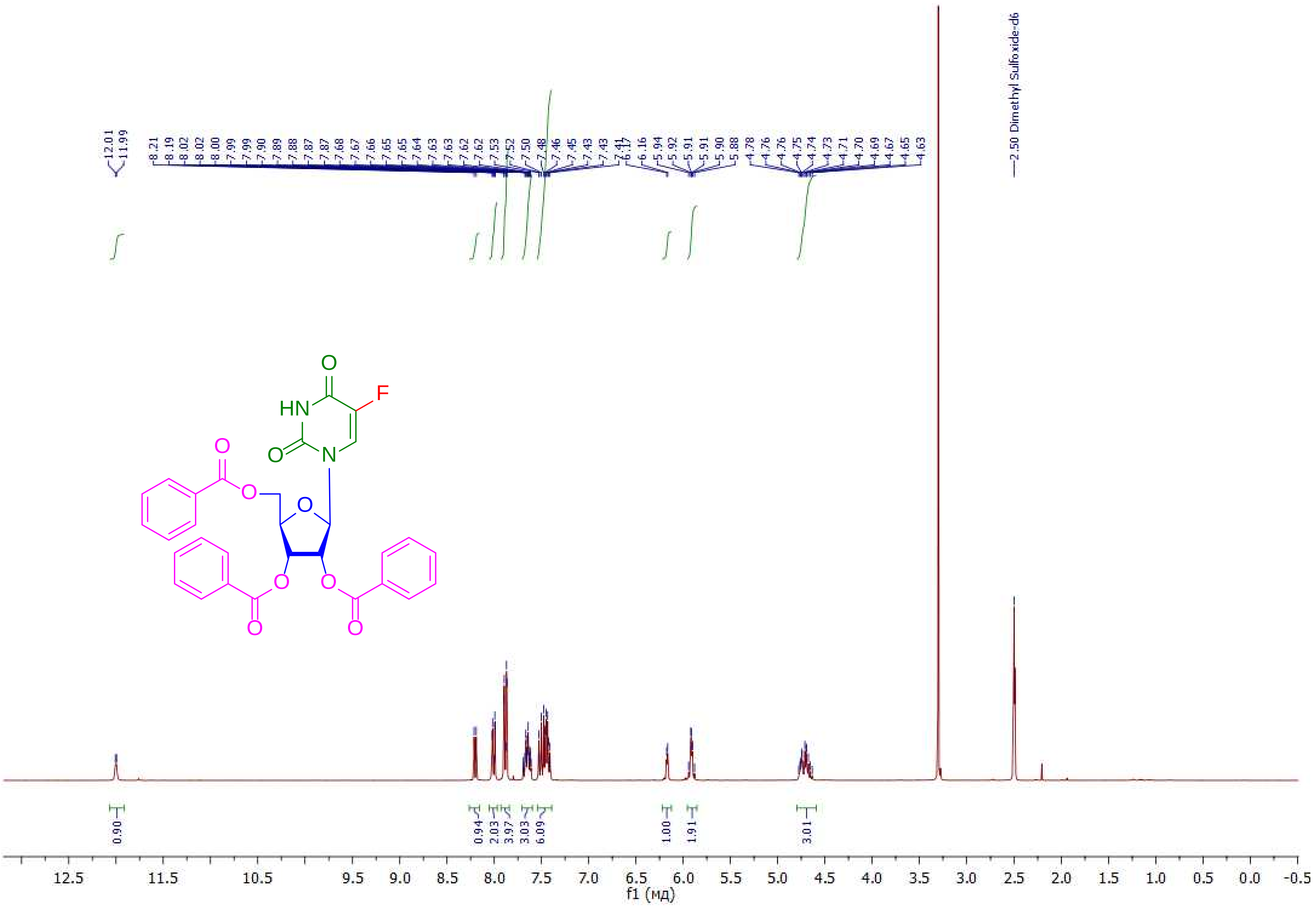

Fig.3. ^{1}H-NMR-spectrum (400 MHz) of 5-fluoro-2′,3′,5′-tri-O-benzoyluridine in DMSO-d_6 at 298 K

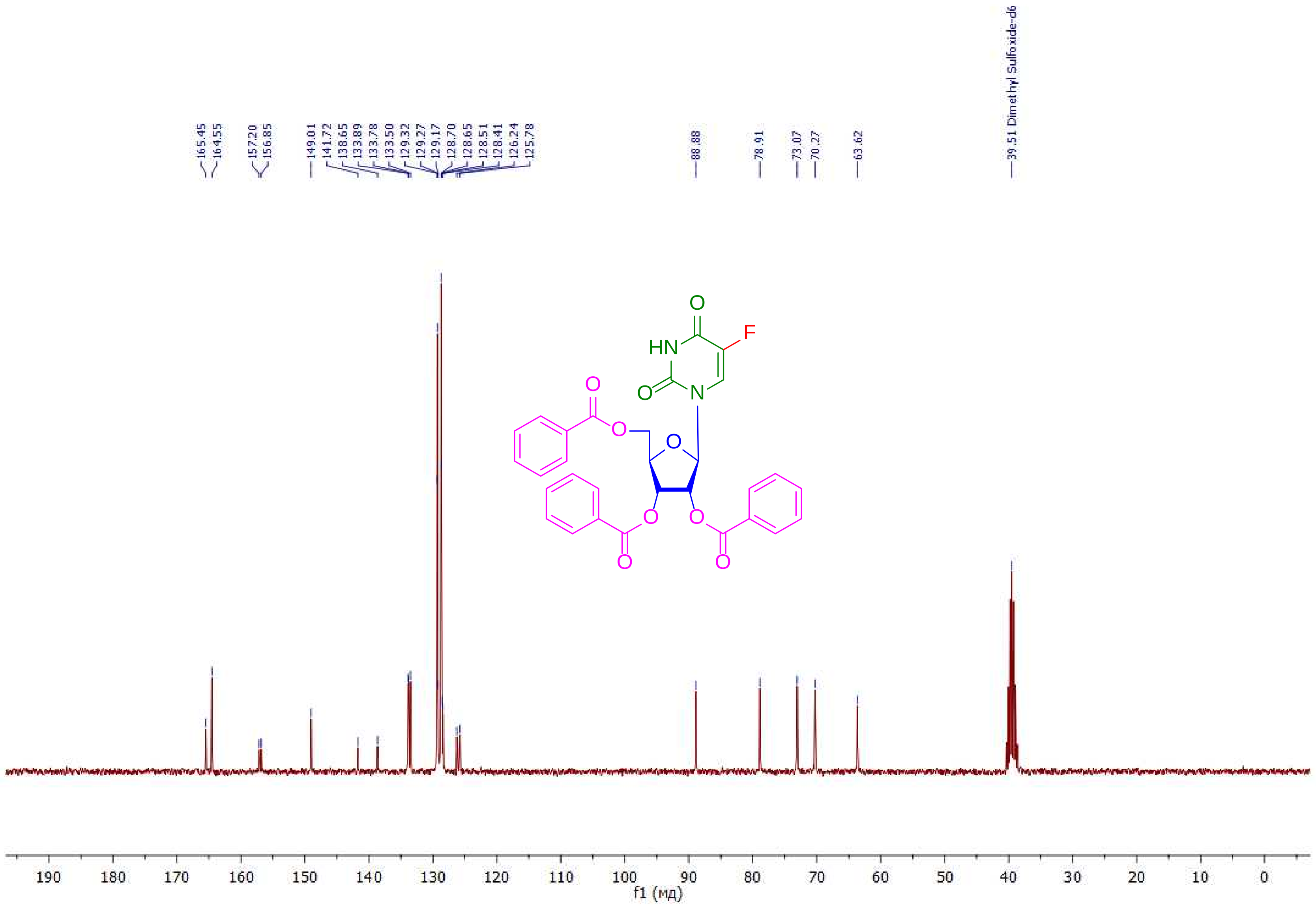

Fig.4. ^{13}C-NMR-spectrum (400 MHz) of 5-fluoro-2′,3′,5′-tri-O-benzoyluridine in DMSO-d_6 at 298 K

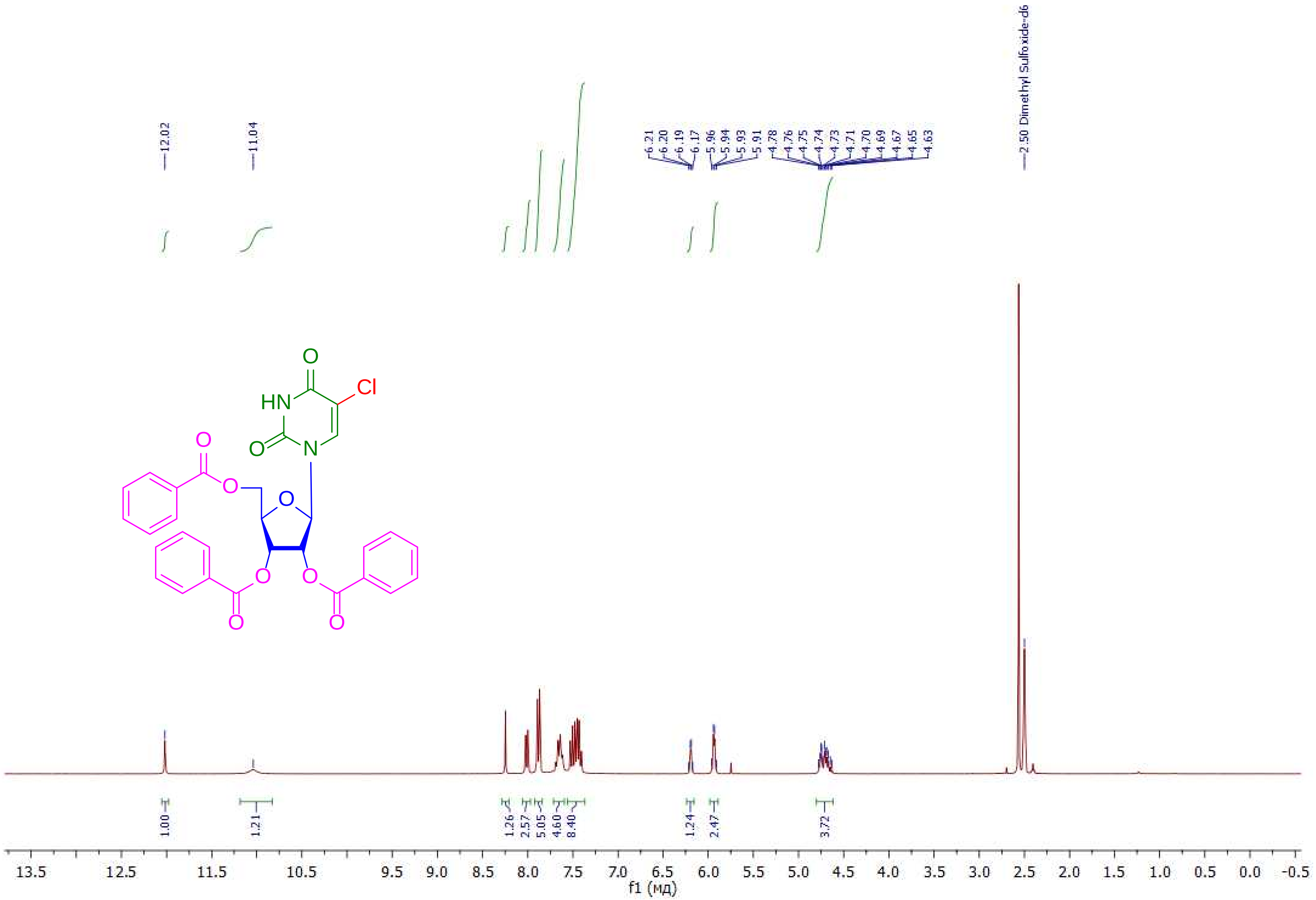

Fig.5. ^{1}H-NMR-spectrum (400 MHz) of 5-chloro-2′,3′,5′-tri-O-benzoyluridine in DMSO-d_6 at 298 K

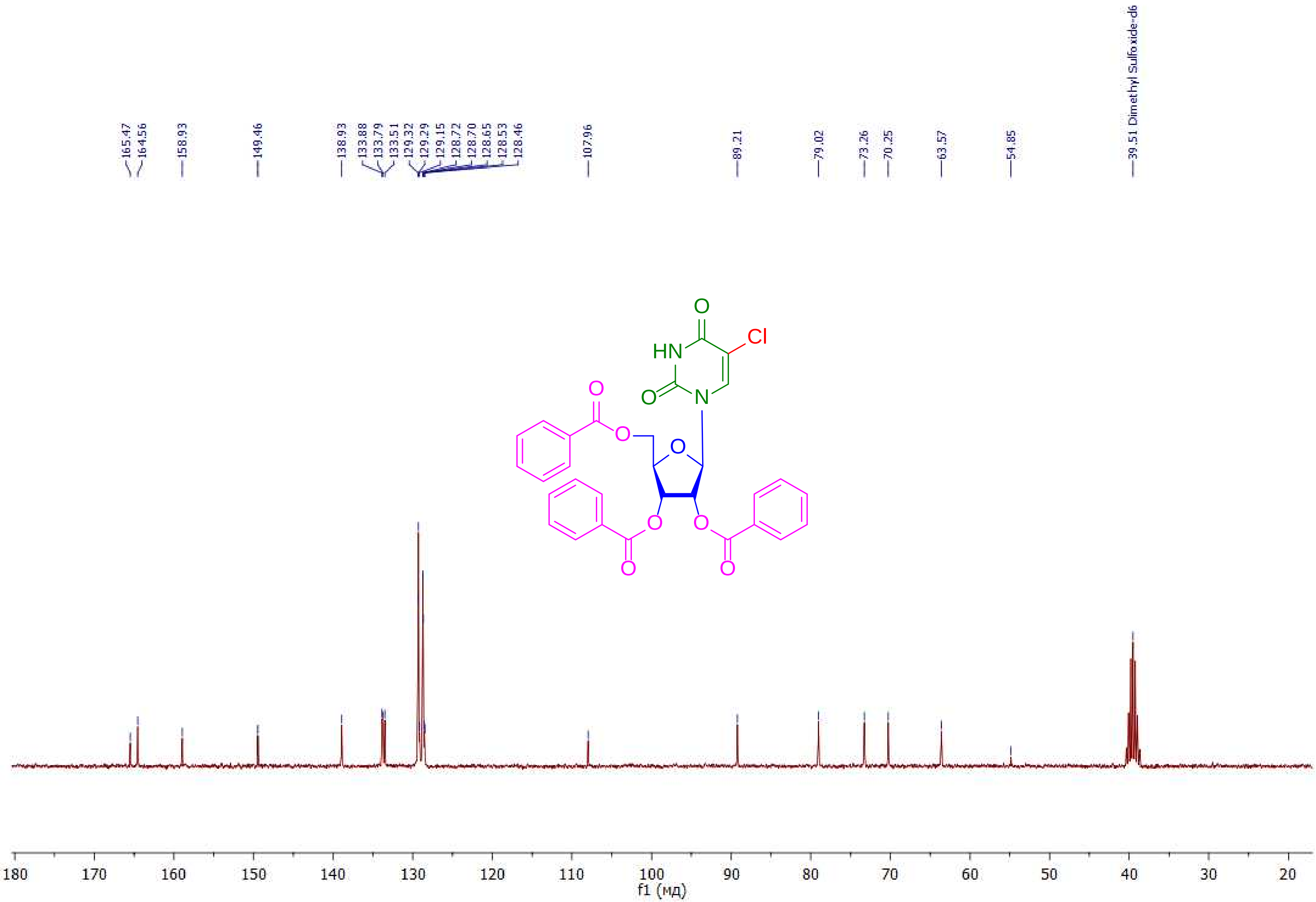

Fig.6. ^{13}C-NMR-spectrum (400 MHz) of 5-chloro-2′,3′,5′-tri-O-benzoyluridine in DMSO-d_6 at 298 K

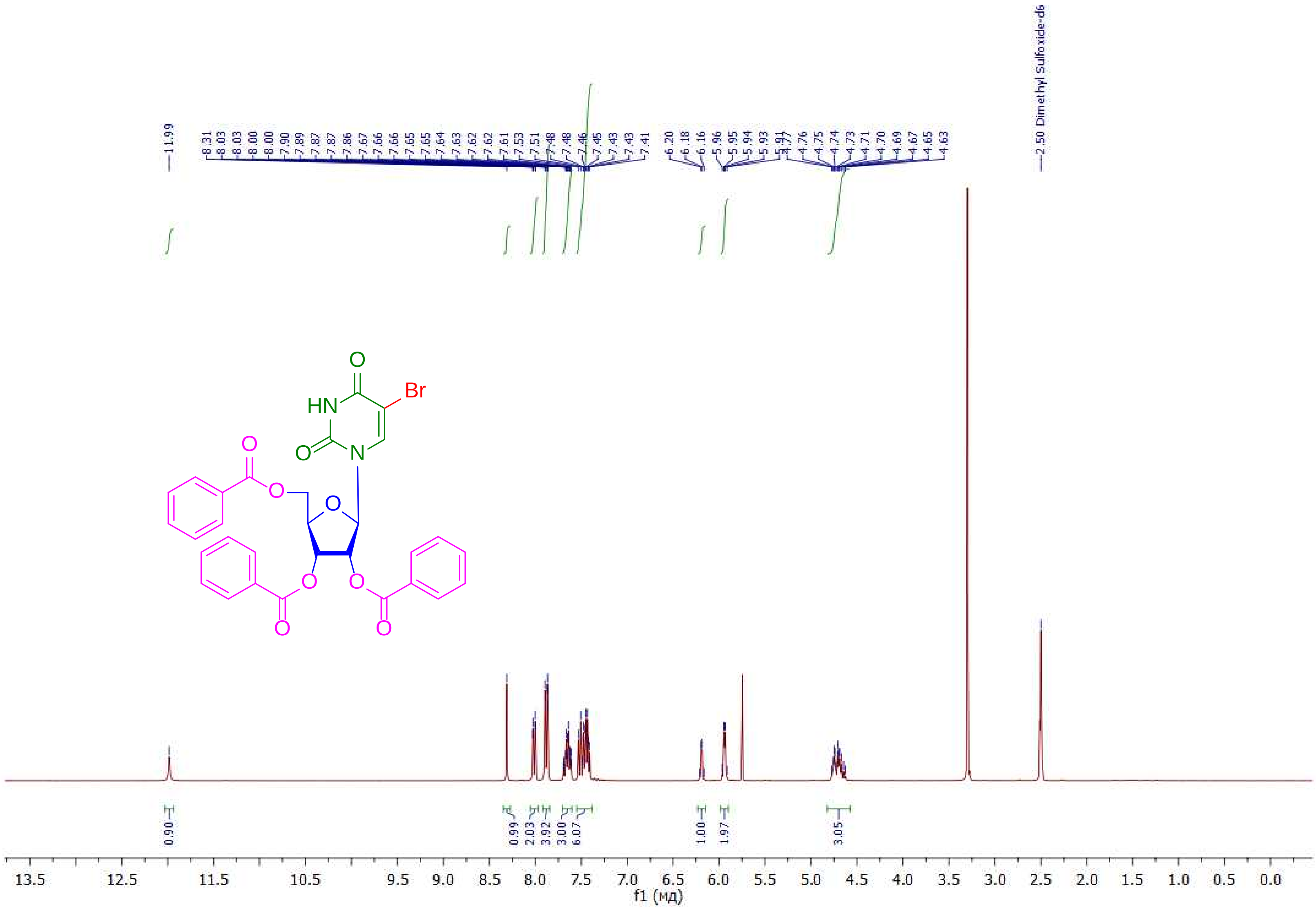

Fig.7. ^{1}H-NMR-spectrum (400 MHz) of 5-bromo-2′,3′,5′-tri-O-benzoyluridine in DMSO-d_6 at 298 K

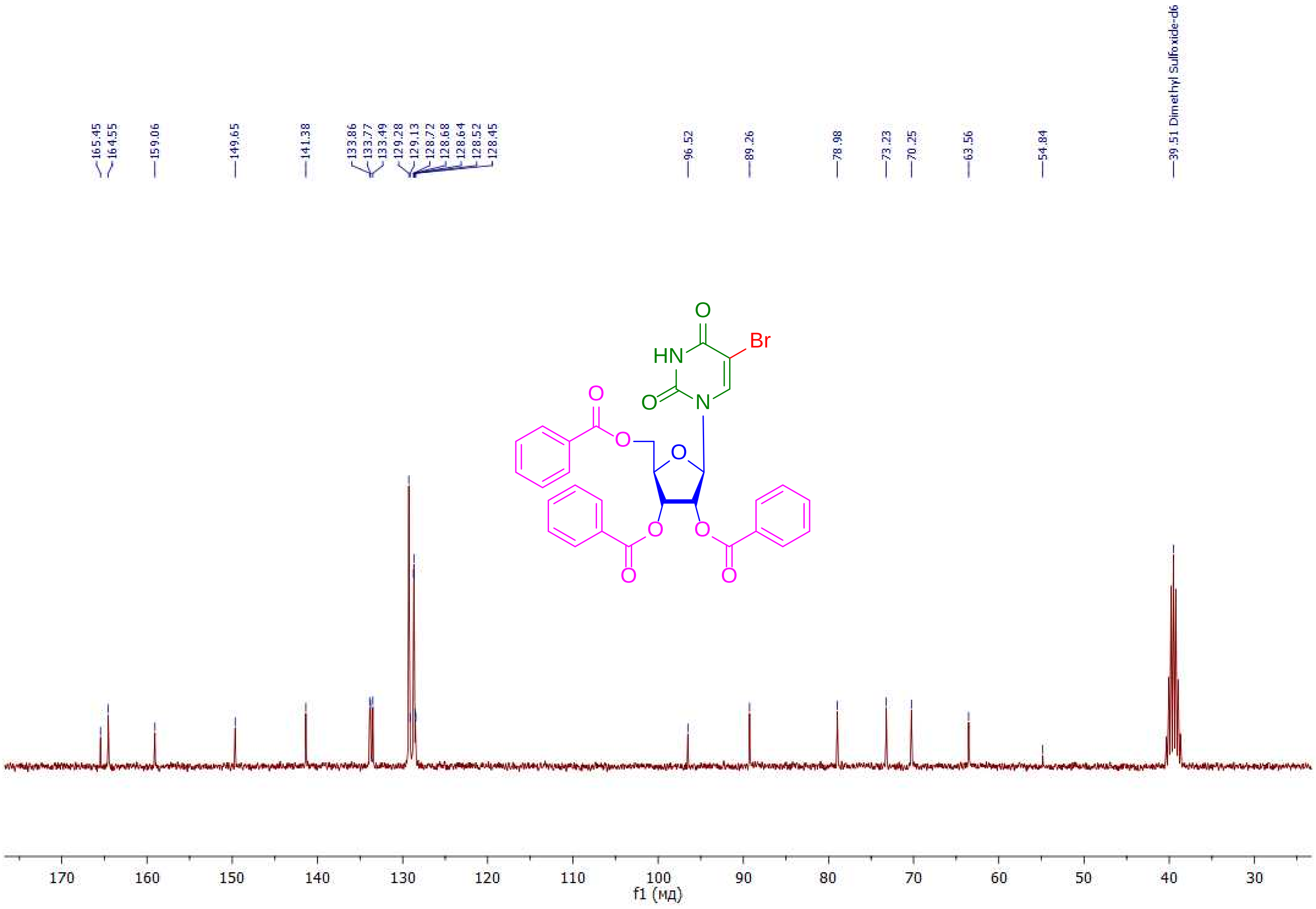

Fig.8. ^{13}C-NMR-spectrum (400 MHz) of 5-bromo-2′,3′,5′-tri-O-benzoyluridine in DMSO-d_6 at 298 K

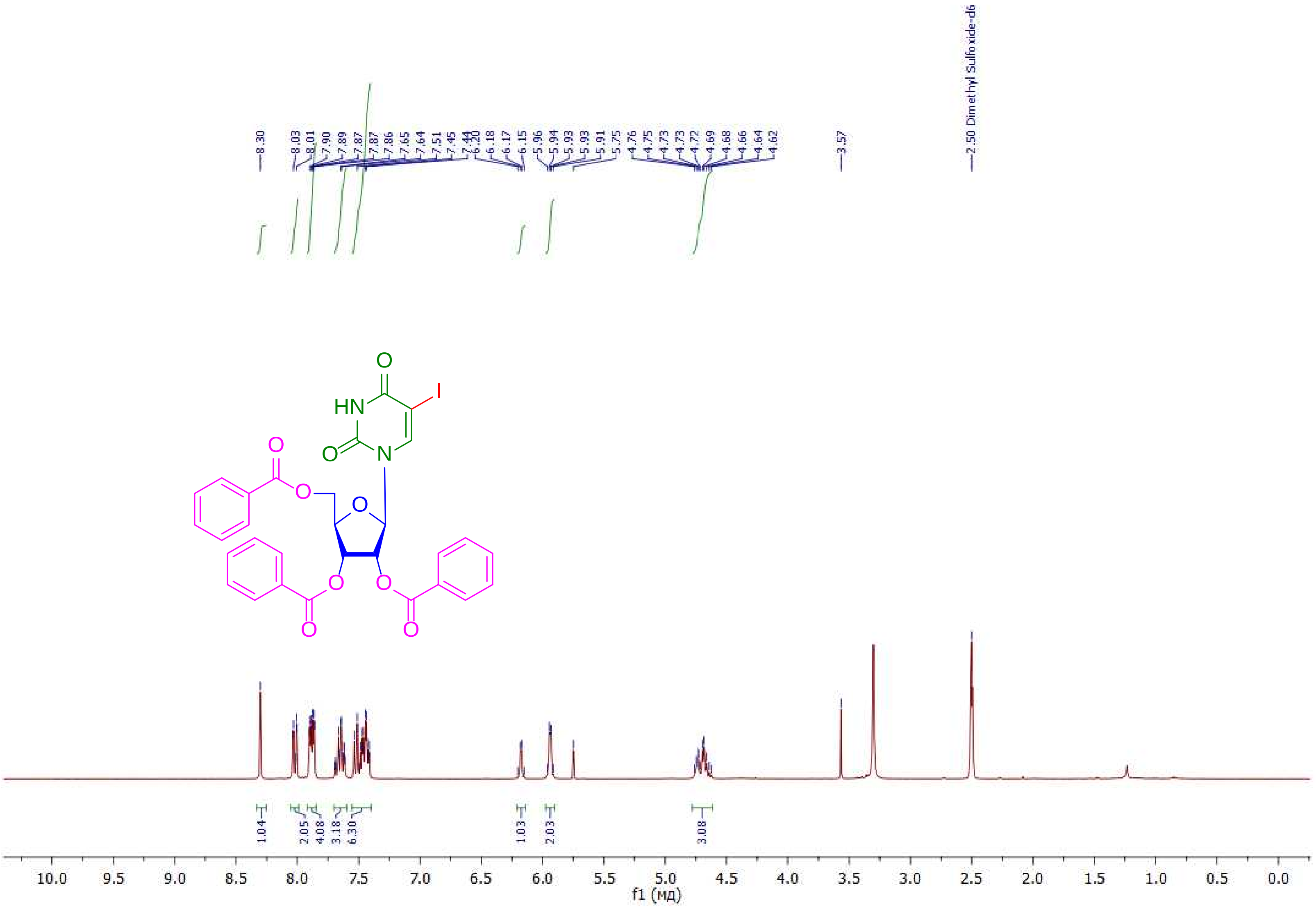

Fig.9. ^{1}H-NMR-spectrum (400 MHz) of 5-iodo-2′,3′,5′-tri-O-benzoyluridine in DMSO-d_6 at 298 K

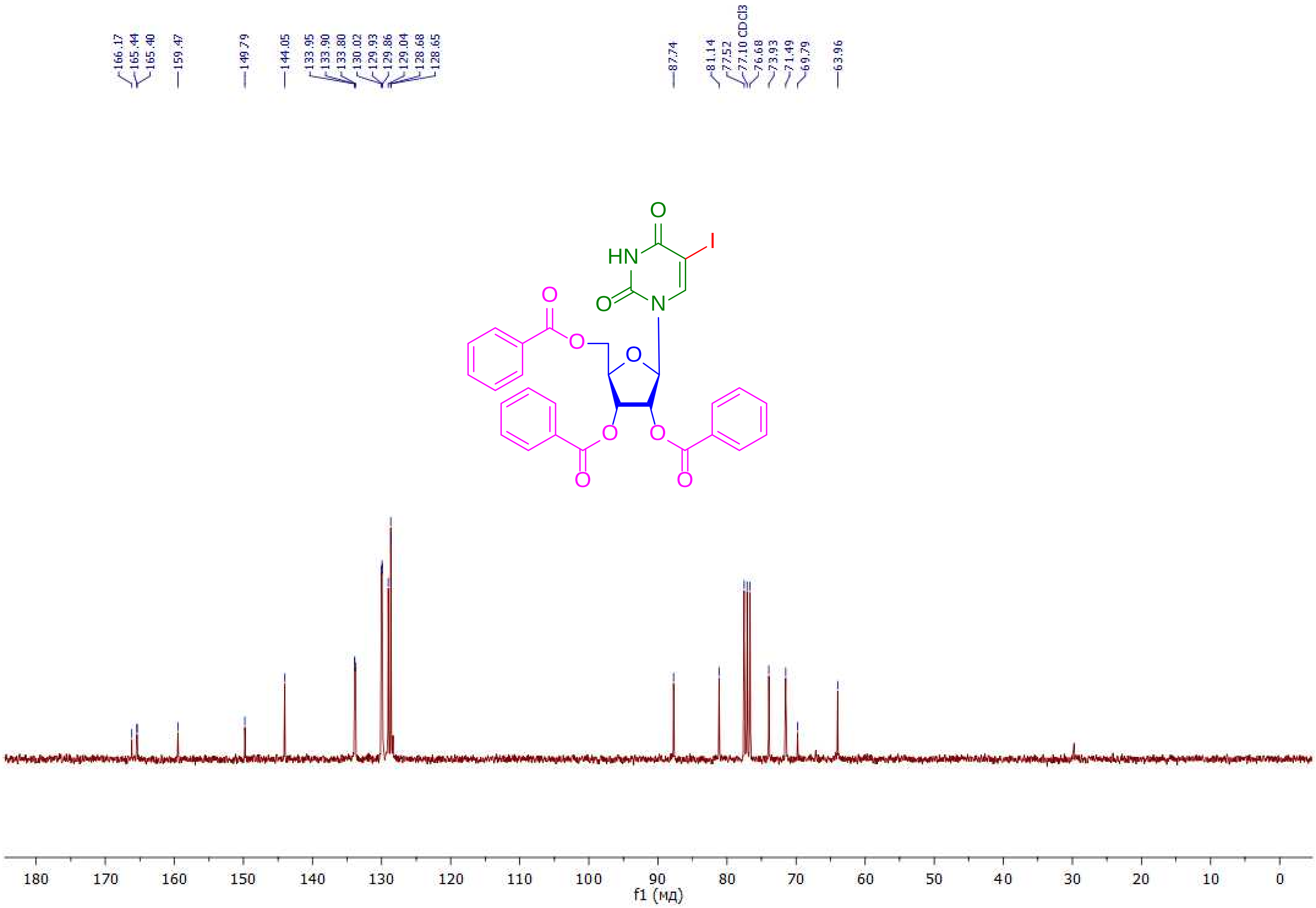

Fig.10. ^{13}C-NMR-spectrum (400 MHz) of 5-iodo-2′,3′,5′-tri-O-benzoyluridine in $CDCl_3$ at 298 K

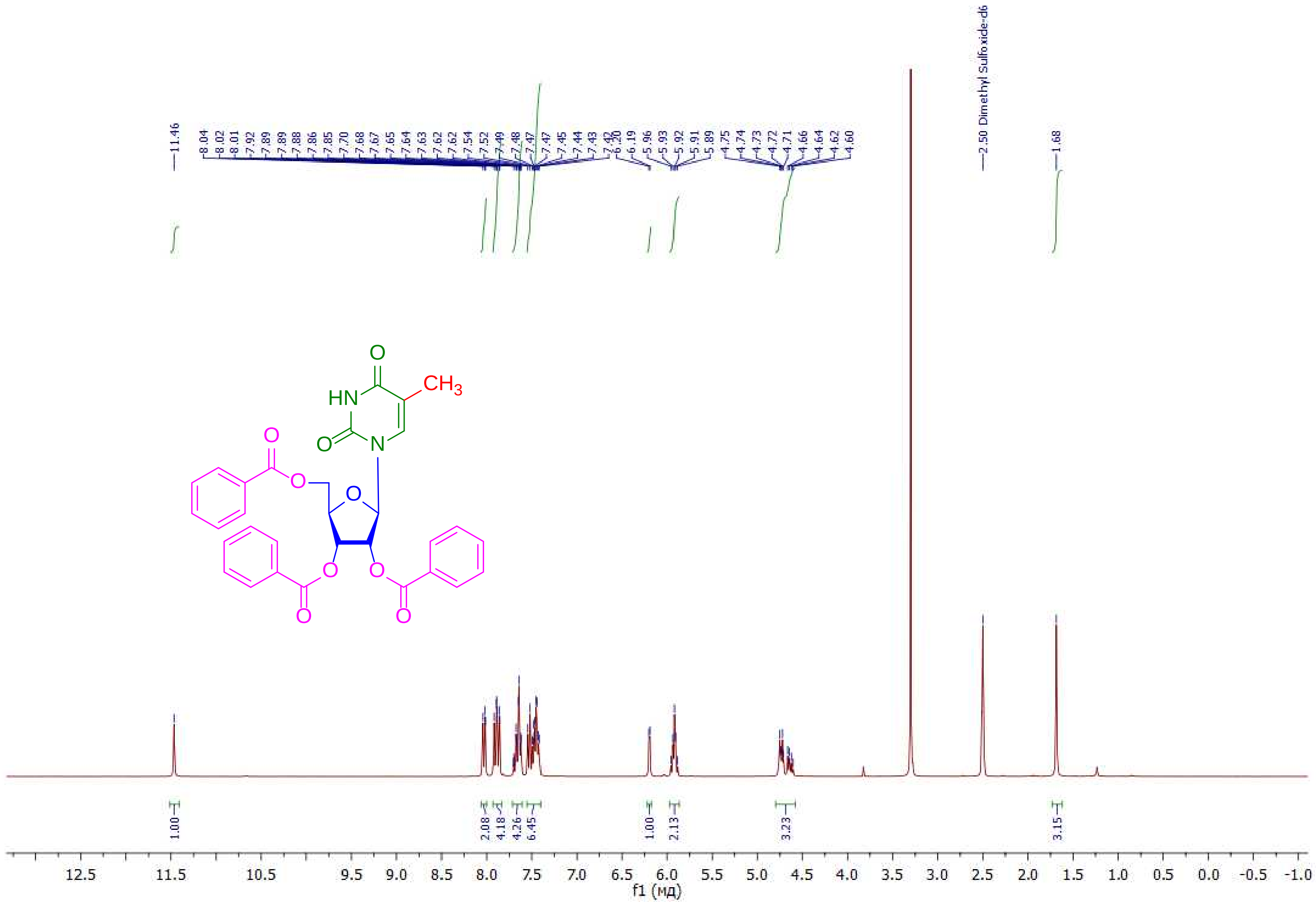

Fig.11. ^{1}H-NMR-spectrum (400 MHz) of 2′,3′,5′-tri-O-benzoylribotimidine in DMSO-d_6 at 298 K

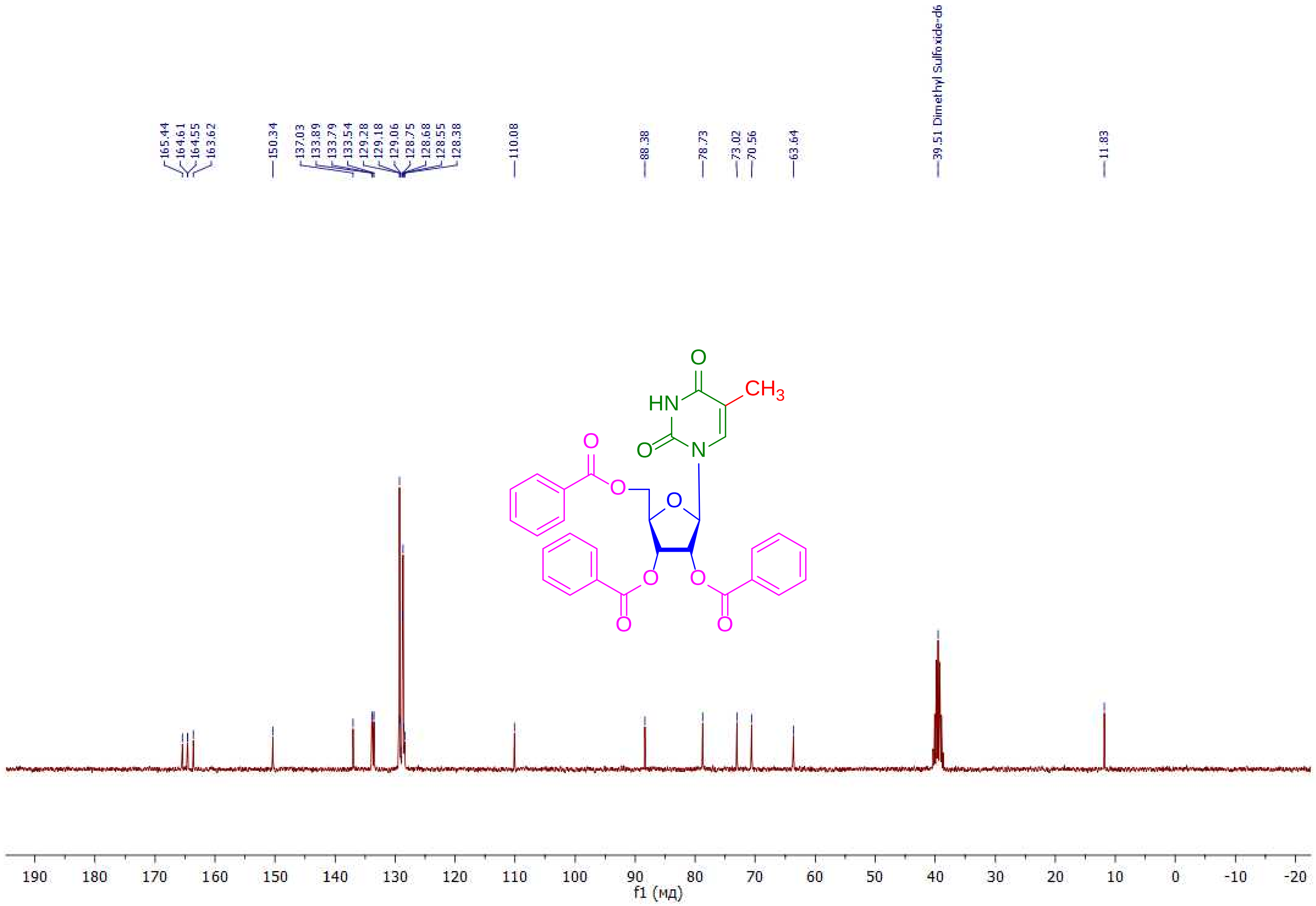

Fig.12. ^{13}C-NMR-spectrum (400 MHz) of 2′,3′,5′-tri-O-benzoylribotimidine in DMSO-d_6 at 298 K

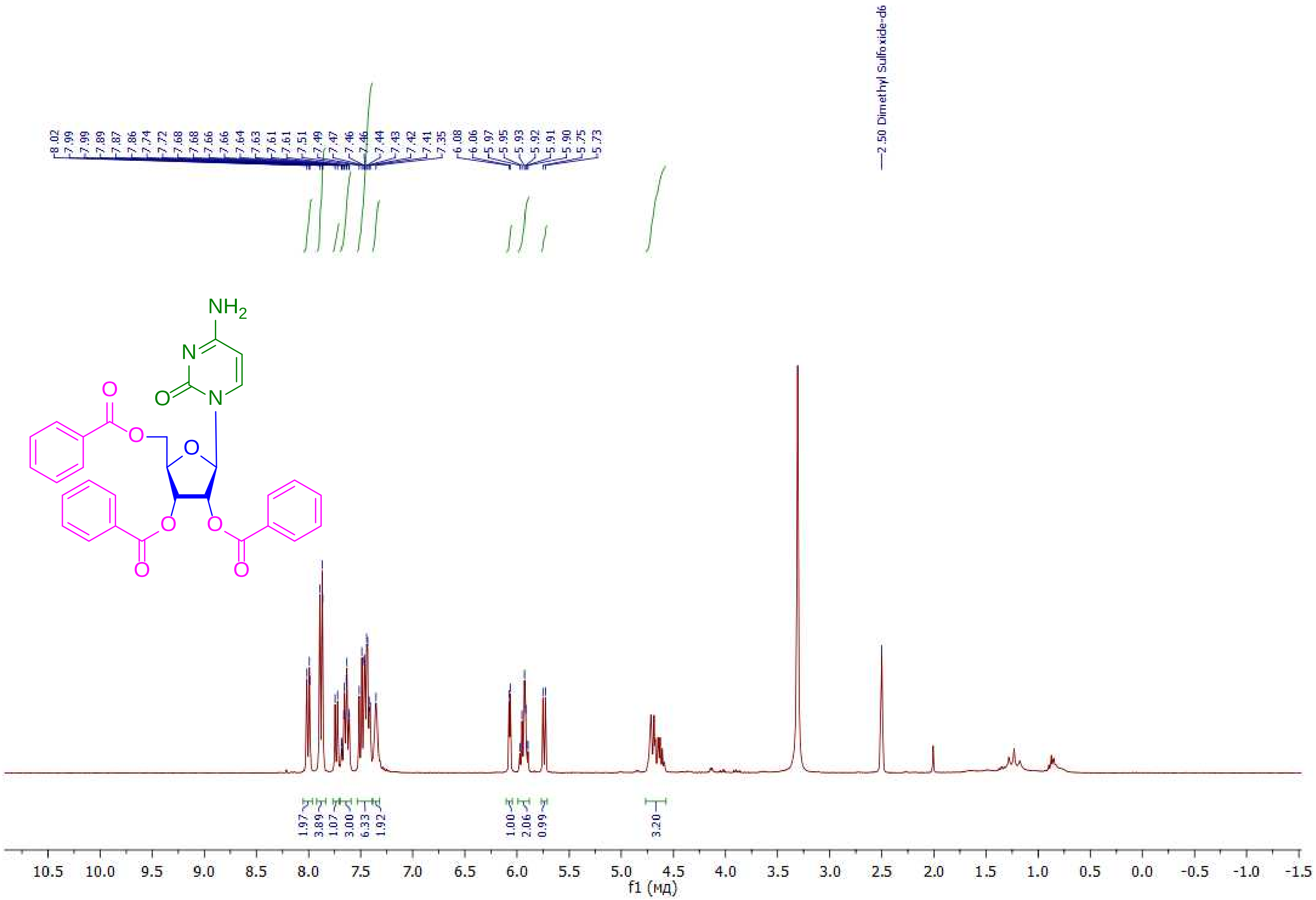

Fig.13. ^{1}H-NMR-spectrum (400 MHz) of 2′,3′,5′-tri-O-benzoylcytidin in DMSO-d_6 at 298 K

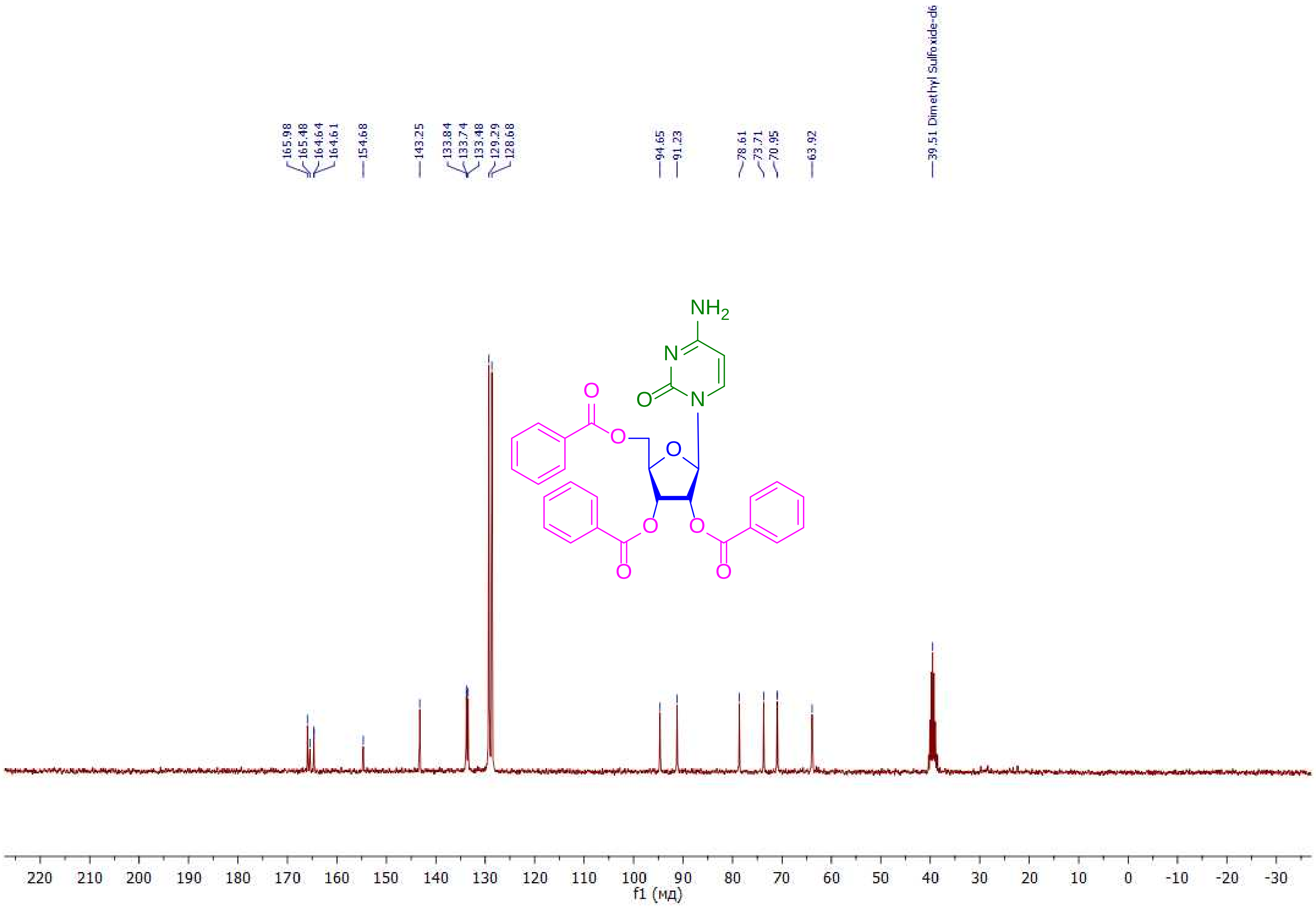

Fig.14. ^{13}C-NMR-spectrum (400 MHz) of 2′,3′,5′-tri-O-benzoylcytidin in DMSO-d_6 at 298 K

Table 1. *Inhibition of Tdp-1 by nucleoside derivatives*.

Cmpd	Compound name	Structure	LogP*	IC_{50} µM	HeLa CC_{50} µM
**1a**	Uridine	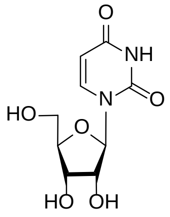	*-2.28*	>50	ND**
**1b**	5-Fluorouridine	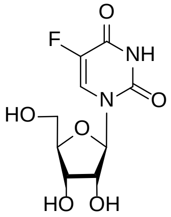	*-2.64*	>50	ND
**1f**	5-Chlorouridine	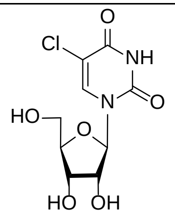	*-2.24*	>50	ND

**1c**	5-Bromoridine	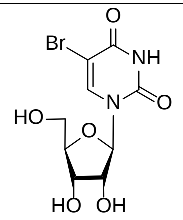	*-1.97*	>50	ND
**1d**	5-Iodouridine	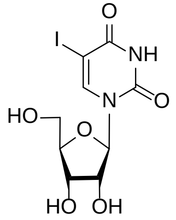	*-1.44*	>50	ND
**1e**	5-Methyluridine (Ribothymidine)	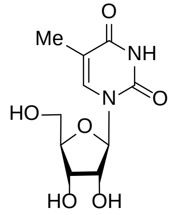	*-1.39*	>50	ND
**2a**	2′,3′,5′-Tri-*O*-benzoyluridine	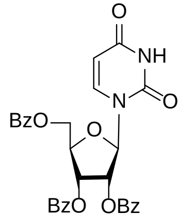	*5.07*	6.3±0.4	ND
**2b**	2′,3′,5′-Tri-*O*-benzoyl-5-fluorouridine	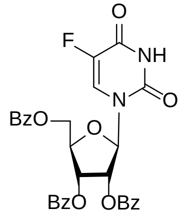	*5.27*	8.5±1.4	>100

**2f**	2′,3′,5′-Tri-*O*-benzoyl-5-chlorouridine	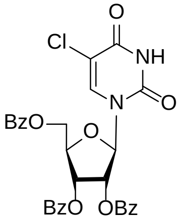	*5.73*	3.6±1.1	>100
**2c**	2′,3′,5′-Tri-*O*-benzoyl-5-bromouridine	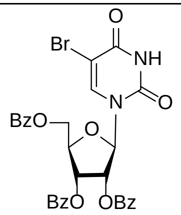	*5.90*	1.5±0.9	>100
**2d**	2′,3′,5′-Tri-*O*-benzoyl-5-iodouridine	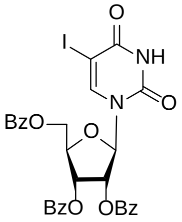	*6.06*	0.6±0.9	>100
**2e**	2′,3′,5′-Tri-*O*-benzoyl-5-methyluridine	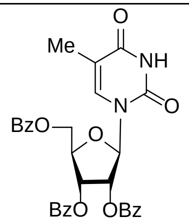	*5.47*	2.7±0.6	>100
**2g**	2′,3′,5′-Tri-*O*-benzoyl-6-methyluridine	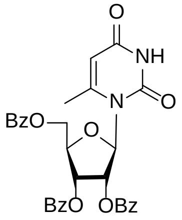	*5.27*	3.4±0.2	>100

**3a**	5′-O-Benzoyluridine	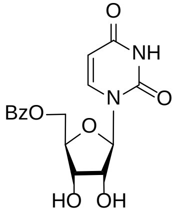	*0.08*	>100	ND
**4a**	2′,3′-Di-*O*-benzoyluridine	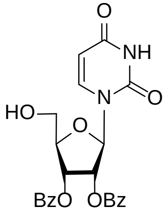	*2.58*	23±6	>100
**5**	1-(2′,3′,5′-Tri-*O*-benzoyl-β-D-ribofuranosyl)-4-pyrimidone	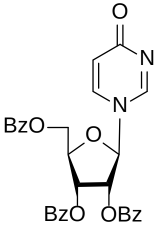	*5.07*	18±1	ND
**6**	1-(2′,3′,5′-Tri-*O*-benzoyl-β-D-ribofuranosyl)-2-pyrimidone	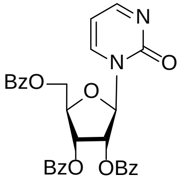	*5.28*	6.0±0.7	>100
**7**	2′,3′,5′-Tri-*O*-benzoyl-2-oxo-4-metoxypyrimidine	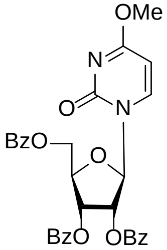	*5.55*	2.91±0.01	>100

**8**	2′,3′,5′-Tri-*O*-benzoyl-cytidine	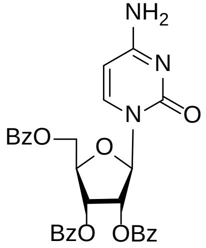	*4.69*	4.3±0.7	~100

* The values of the partition coefficient of the compounds between the 1-octanol-water phases (logP) were calculated using the Instant J. Chem. (ChemAxon®)

**ND – not determined

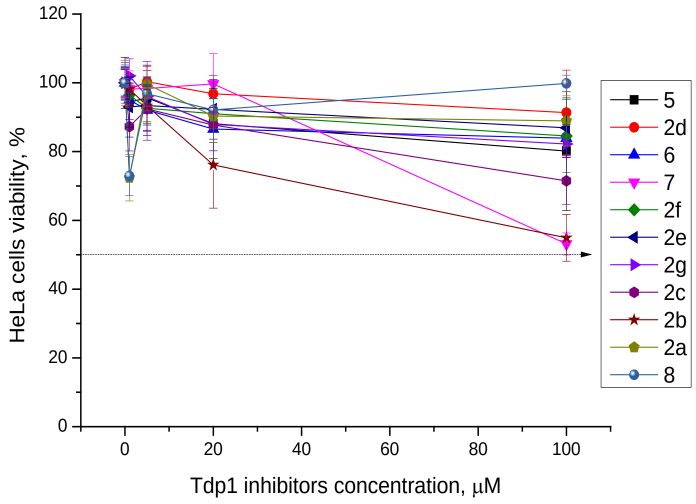

Figure 1S. Dose-dependent action of the lipophilic pyrimidine nucleosides on HeLa cell viability measured in the EZ4U assay.

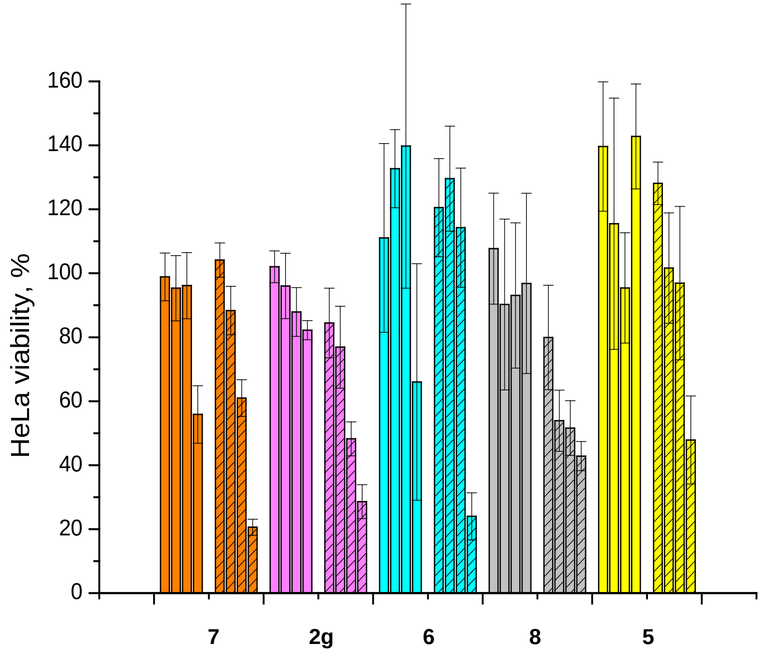

(a)

Figure 2S. Dose-dependent action of the lipophilic nucleosides in combination with topotecan (Tpc) on HeLa cell viability in the EZ4U assay. The unshaded histogram bars denote cell viability in the presence of a single Tdp1 inhibitor. The hatched histogram bars indicate cell viability in the presence of a combination of a Tdp1 inhibitor with 2 μM topotecan. **(a)** Compounds showing synergy with topotecan. **(b)** Inactive compounds. Each of the four bars corresponds to an inhibitor concentration (from left to right) of 1, 5, 20, and 100 μM.

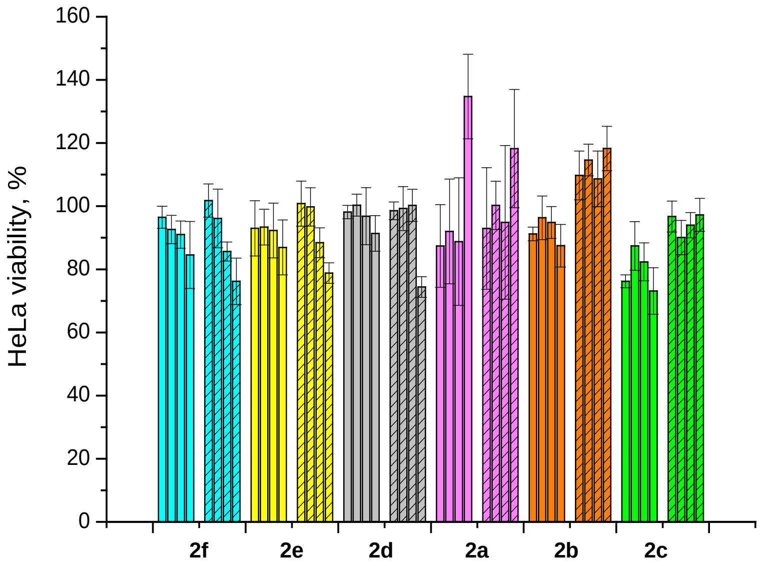

(b)

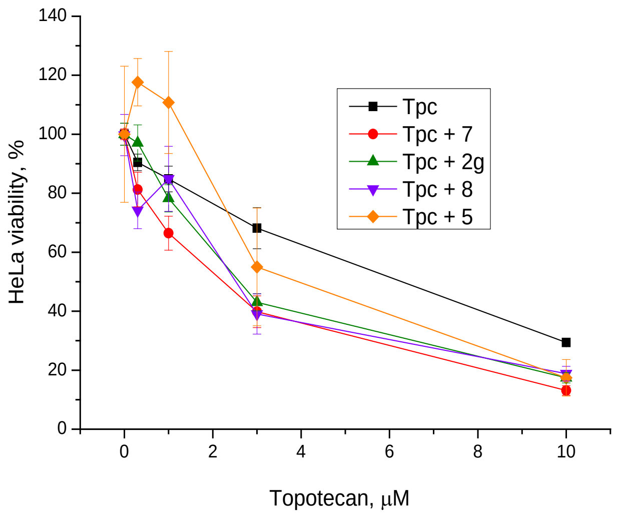

Figure 3S. Dose-dependent action of topotecan (Tpc) in combination with one of the nucleoside derivatives on HeLa cell viability in the EZ4U assay.

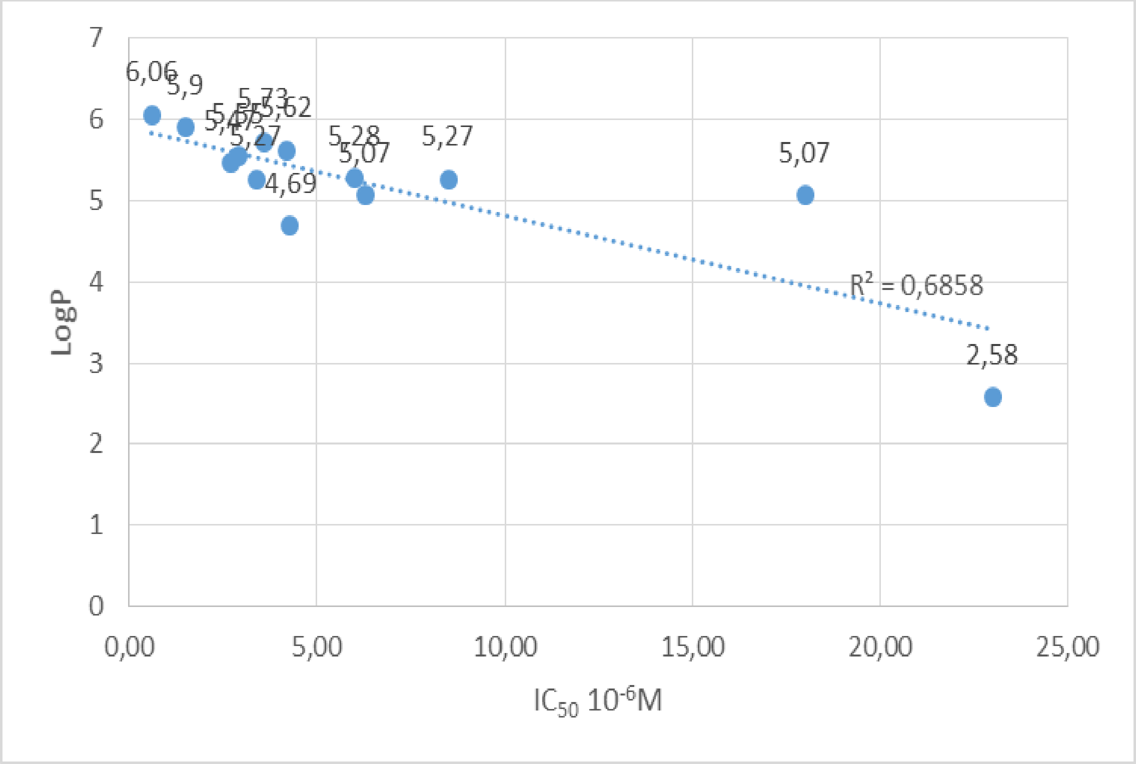

Figure 4S. Correlation diagram between Tdp-1 inhibition IC_{50} and logP of nucleoside derivatives for compounds **2a**-**2g**, **4a**, 5-**9**.

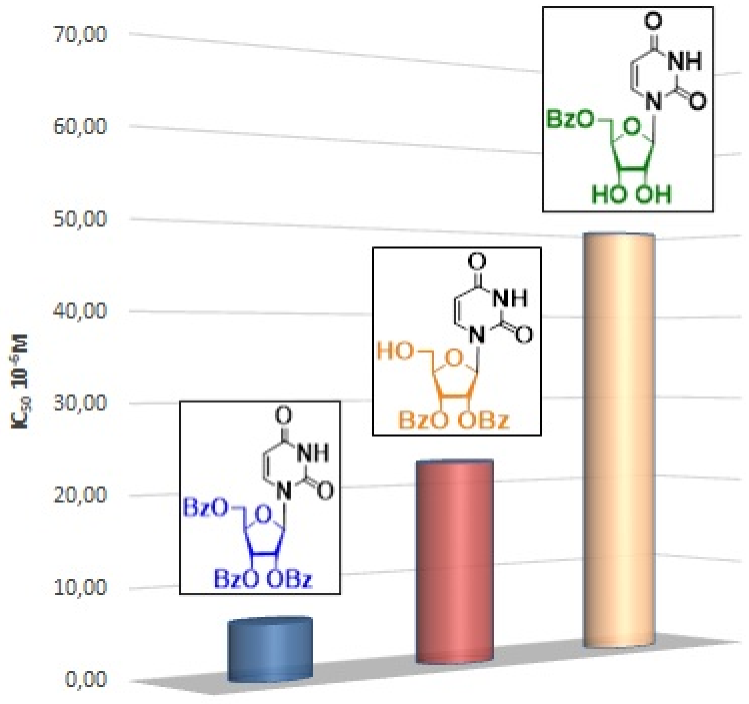

Figure 5S. Structure-cativity relationship between Tdp-1 inhibition and the quantity of benzoyl groups in nucleoside ribofuranose moiety.
